# Role of Transforming Growth Factor β in Uterine Fibroid Biology

**DOI:** 10.3390/ijms18112435

**Published:** 2017-11-17

**Authors:** Michał Ciebiera, Marta Włodarczyk, Małgorzata Wrzosek, Błażej Męczekalski, Grażyna Nowicka, Krzysztof Łukaszuk, Magdalena Ciebiera, Aneta Słabuszewska-Jóźwiak, Grzegorz Jakiel

**Affiliations:** 1 First Department of Obstetrics and Gynecology, The Centre of Postgraduate Medical Education, 00-416 Warsaw, Poland; as.jozwiak@op.pl (A.S.-J.), grzegorz.jakiel1@o2.pl (G.J.); 2 Department of Biochemistry and Clinical Chemistry, Department of Pharmacogenomics, Medical University of Warsaw, 02-097 Warsaw, Poland; mdwlodarczyk@gmail.com (M.W.); malgorzata.wrzosek@wum.edu.pl (M.W.); grazyna.nowicka@wum.edu.pl (G.N.); 3 Department of Gynecological Endocrinology, Poznan University of Medical Sciences, 60-513 Poznan, Poland; blazejmeczekalski@yahoo.com; 4 Department of Obstetrics and Gynecological Nursing, Faculty of Health Sciences, Medical University of Gdansk, 80-210 Gdansk, Poland; krzysztof.lukaszuk@invicta.pl; 5 INVICTA Fertility and Reproductive Center, 80-172 Gdansk, Poland; 6 Students’ Scientific Association at the I Department of Obstetrics and Gynecology, Medical University of Warsaw, 02-015 Warsaw, Poland; mciebiera93@gmail.com

**Keywords:** uterine fibroid, leiomyoma, transforming growth factor β, pathophysiology, therapy

## Abstract

Uterine fibroids (UFs) are benign tumors of the female genital tract made of the smooth muscle of the uterus. UF growth depends mostly on the influence of the steroid hormones and selected growth factors. Transforming growth factor β (TGF-βs) is a polypeptide that consists of three isoforms: TGF-β1, TGF-β2, and TGF-β3. At present, TGF-β is considered to be one of the key factors in the pathophysiology of UFs. It plays a major role in cellular migration within the tumor, stimulates tumor growth, and enhances tumor metabolism. As a consequence of various dependencies, the synthesis and release of TGF-β in a UF tumor is increased, which results in excessive extracellular matrix production and storage. High concentrations or overexpression of TGF-β mediators may be responsible for clinically symptomatic UFs. The aim of this review was to check the available evidence for the influence of the TGF-β family on UF biology. We conducted their search in PubMed of the National Library of Medicine with the use of the following selected keywords: “uterine fibroid”, “leiomyoma”, and “transforming growth factor β”. After reviewing the titles and abstracts, more than 115 full articles were evaluated. We focused on the TGF-β-related molecular aspects and their influence on the most common symptoms that are associated with UFs. Also, we described how the available data might implicate the current medical management of UFs.

## 1. Introduction

Uterine fibroids (UFs) are benign tumors of the female genital tract that are made of the smooth muscle of the uterus. They are a very common pathology, affecting up to 80% of women in some populations [1,2], and may impair normal functioning and lower the quality of patient life [2,3]. The symptoms, especially in women of childbearing age, include iron deficiency anemia, abdominal pain, pelvic pain, or gastrointestinal symptoms manifested by constipation or bloating [1,4]. UFs are also one of the recognized factors negatively affecting fertility [5,6]. All tumors can create an unfavorable environment for implantation and cause various types of homeostatic disorders that affect normal development of the embryo, whereas large tumors may disturb the anatomy of the reproductive tract, not allowing for fertilization or pregnancy growth [5,6,7]. In postmenopausal women, UFs cause compression symptoms, but abnormal spotting or bleeding are most often reported [1,2,8].

Both, direct and indirect costs that are associated with the treatment of UFs are considerable and result in significant health care expenditures. Thus, researchers all over the world are constantly searching for new solutions as far as UF therapies are concerned [9,10,11].

### 1.1. Transforming Growth Factor β—Signaling Pathways, Proliferation and Fibrosis

Cytokines are low-molecular-weight proteins, which are produced by the immune system, which predominantly act in a paracrine/autocrine manner [12]. Cytokines affect tumor biology, influence the growth and survival of UF cells, regulate angiogenesis, and shape the extracellular matrix (ECM) [13,14,15]. Cytokines may be responsible for UF-associated pain, infertility, or obstetric pathologies [16].

**Figure 1:**
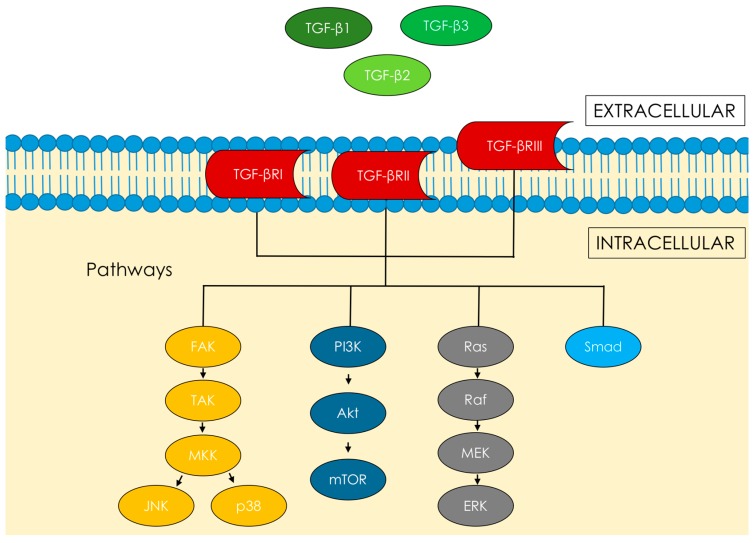
TGF-β isoforms, TGF-β receptors; TGF-β intracellular signaling pathways. TGF-β: transforming growth factor β; FAK: focal adhesion kinase; TAK: TGF-β-activated kinase; MKK: Mitogen-activated protein kinase kinase; JNK: c-Jun N-terminal kinase; p38: p38 mitogen-activated protein kinases; PI3K: Phosphoinositide 3-kinase; Akt: Protein kinase B; mTOR: mechanistic target of rapamycin; Ras: Ras protein; Raf: Raf protein; MEK: MAPK/ERK kinase; ERK: Extracellular signal-regulated kinases; Smad: Smad protein.

Although numerous cytokines are involved in UF biology, transforming growth factor β (TGF-β) appears to be one of the most important myometrium-associated cytokines. It controls the proliferation and differentiation in most types of human cells [17], and is well-known in diseases that are connected with abnormal or uncontrolled fibrosis like myocarditis, nephropathy, bowel inflammatory diseases, etc. [13,14,18,19]. TGF-β is a polypeptide consisting of three isoforms (TGF-β1, TGF-β2, and TGF-β3), which have their own pathway for transmembrane receptors, TGF-βR-I and TGF-βR-II [20] (Figure 1).

The TGF-β family is responsible for the modulation of paracrine and autocrine factors of inflammation, cell cycle, and growth [13,14,21]. TGF-β is known as a potent chemoattractant for macrophages and fibroblasts [12]. It inhibits the division of some cells, induces apoptosis, and also affects the development of ECM [13,22,23].

Various researchers have confirmed the presence of different TGF-β isoforms in the pathophysiology of UFs [24,25], which is the key factor in cellular migration within the tumor, stimulates tumor growth, and enhances tumor metabolism. TGF-β regulates the expression and growth of uterine smooth muscle and UFs. TGF-β expression in the smooth muscle of the uterus, which is in direct contact with the fibroid, is significantly increased, as has been demonstrated in laboratory studies [26,27]. It has been shown that the expression of TGF-β in UF tissue as compared to the control group (normal smooth muscle) is almost twice as high [28]. Interestingly, the TGF-β3 isoform occurs in UF tissue at concentrations almost five times higher than in the healthy myometrium [14,29].

The role of TGF-β signaling in the development of UFs is complex [1,2,30]. Different isoforms of TGF-β and their receptors are expressed in the human myometrium and UF tumors [12,13,31]. In normal smooth muscle cells, TGF-β acts as a potent tumor suppressor through growth inhibition and stimulation of apoptosis. On the other hand, overexpression of TGF-β in UFs is also observed and appears to play an important role in their growth and symptom progression [13,24]. The role of the TGF-β family in the pathogenesis of UF has been proven in in vitro studies. However, data on the major sites of action of TGF-β and their impact on the risk for UFs remain limited.

### 1.2. Extracellular Matrix

Chronic inflammation is described as the migration of inflammatory cells to exact locations and an increased expression of proinflammatory mediators and factors in longer periods of time [19]. The fibrotic reaction of the connective tissue is mainly characterized by an increased production of ECM and the accumulation of the mesenchymal cell component [19]. Normal ECM undergoes a continuous and balanced turnover process, which contributes to maintaining its proper amount and stiffness. Matrix enzymes are regulated by special inhibitors of extracellular matrix metalloproteinases (MMPs) (tissue inhibitor of metalloproteinases, TIMP) [32].

A uterine fibroid is in fact a mass of tumor cells embedded in a large amount of ECM [33]. The ECM building the fibroid is much more abundant than in the well-formed myometrium tissue. It is believed that in case of a UF tumor, the ECM volume may be over twice the volume of that found in the healthy myometrium. Collagen (especially types I and III), fibronectin, and proteoglycans are the main components of ECM [17]. Collagen fibers that are found in the UFs have a distorted spatial structure and differ from their counterparts in normal tissues [17,34,35].

**Figure 2:**
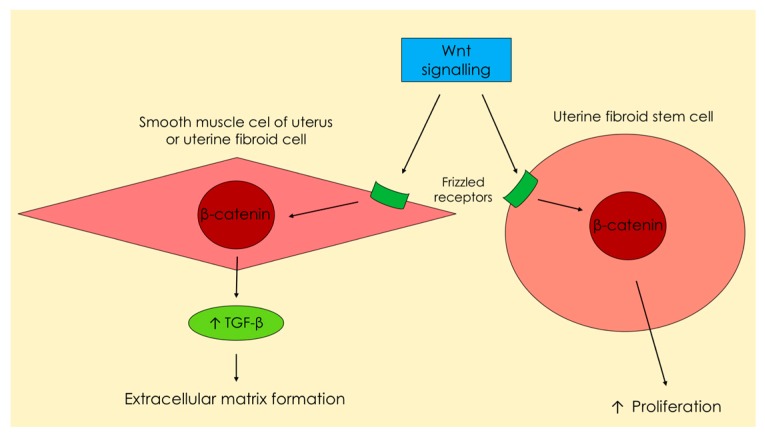
Increased secretion of Wnt ligands under the influence of estrogen and progesterone (Figure 3) from smooth muscle cells, which are placed around uterine fibroid stem cells. This pathway leads to excessive production of the transforming growth factor β and extracellular matrix, as well asenhanced proliferation of uterine fibroid stem cells.

As a consequence of various dependencies, the synthesis and release of TGF-β in a UF tumor is increased, which results in excessive ECM production and storage [17,35] (Figure 2).

It should be emphasized that, out of all the TGF-β isoforms, the TGF-β3 isoform was found to be one of the main inductors of the elevated production of ECM and decreased production of ECM degradation factors in UFs [13,14,29].

### 1.3. Regulation by Steroids

**Figure 3:**
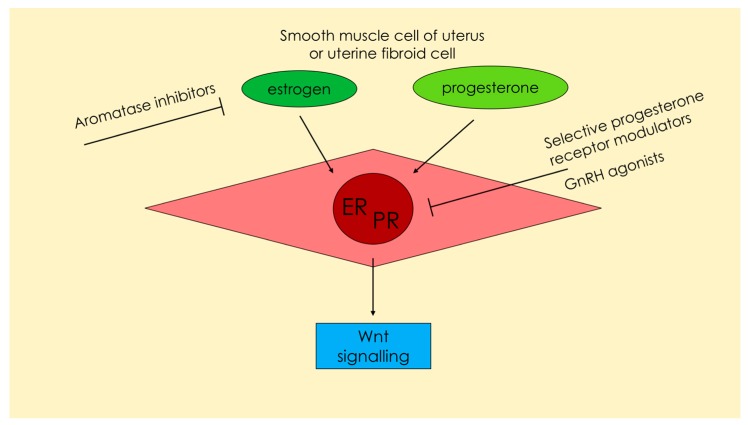
Estrogen and progesterone—two main hormones influencing the metabolism and proliferation of uterine smooth muscle cells or uterine fibroid cells. Wnt signaling is one of the most important pathways in uterine fibroid pathophysiology. Excessive production of transforming growth factor β depends greatly on Wnt signals. The drugs influencing the hormonal pathways and their potential site of effect are presented. ER: Estrogen receptor; PR: Progesterone receptor.

According to the available data, UF growth depends mostly on the influence of the steroid hormones [24,36,37] (Figure 3).

Estrogen and progesterone concentrations vary, depending on patient age. UFs are not observed in pre-pubescent girls, which indicates that they depend on the hormonal changes during that period [38] (Figure 3). Estrogens play an important role in the pathophysiology of UFs. However, a large group of researchers consider progesterone to be the main factor initiating uterine muscle differentiation and its subsequent abnormal growth [24]. Various studies have confirmed that progesterone activates cell division within the smooth muscle of the uterus, especially in the second phase of the cycle when progesterone concentrations are significantly elevated [36,39]. The main mechanism of action of progesterone in tumorigenesis of UFs is its effect on the increase in the concentration of selected growth factors (Figure 3). These hormone-dependent factors are secreted and act directly on the muscle, making it a self-stimulating growth process [13,24].

### 1.4. Genetics

The available literature reports suggest the existence of additional factors, which are a part of the complex chain of interactions resulting in the appearance and growth of UFs [24]. Genetic studies show that a uterine fibroid is a monoclonal neoplasm stemming from a primary cell which has undergone specific molecular changes [40,41]. The first link in this chain of pathogenesis appears to be the presence of a modified myometrial cell, which has the capacity for uncontrolled division and unrestricted growth [24,40,41]. The transformed cell needs adequate stimuli to divide and produce ECM [24]. The exact cause of the development of UFs remains unclear, but there is evidence that they may be the result of a combination of genetic, hormonal, and environmental factors [1,24,42]. Until recently, the number of publications showing a specific location in the genome associated with UF occurrence has been scarce. 2011 has been heralded as a breakthrough year for UF genetics, when Makinen and colleagues discovered the potential genetic background of UFs—mutations were detected within the gene encoding the mediator complex subunit 12 (MED12) [43], and these molecular changes apply also to TGF-β [24].

### 1.5. TGF-β and Implications for Therapy

The abovementioned observations can be translated into clinical management. Estrogen and progesterone have been shown to upregulate the expression of multiple angiogenic factors in the myometrial and UF tissues [13,22]. That is why application of different hormonal agents may exert their effects through the inhibition of angiogenesis in tumors [44,91]. The main substances, which are or may be used in the treatment of UFs, and which affect the pathways dependent on TGF-β, are gonadotropin-releasing hormone analogs (GnRHa), aromatase inhibitors (AIs) [45], or selective progesterone receptor modulators (SPRMs) [46]. Their exact role and pathways are explained later in the text.

### 1.6. Future Ideas

Early prevention, adequate prophylaxis, and timely treatment of UFs in women from high-risk groups continue to be our priorities. The ideal methods of prevention and early-stage therapy should be inexpensive and relatively risk-free. Current therapies, such as myomectomy, hysterectomy, embolization, GnRHa, and SPRMs are relatively effective as far as treatment and prevention of the onset of symptoms are concerned, but are expensive or associated with complications or side effects [2,46]. Clearly, all of the pathways that affect the TGF-β family, especially the TGF-β3 isoform, will have the potential to translate into further work, particularly in the field of pharmacology.

If the development of UFs is considered as a TGF-β-dependent proliferation process, the treatment should be sought among antiproliferative and antifibrotic therapies. The available clinical studies demonstrated that some substances like cabergoline [47], epigallocatechin gallate [48], or vitamin D [49,50], might be useful in the pharmacological treatment of UFs [51].

The aim of this review is to summarize the current literature reports regarding the role of the TGF-β family in the UF biology. We focused on the TGF-β-related molecular aspects and their influence on the most common symptoms that are associated with UFs. Also, we described how the available data might implicate the current medical management of UFs.

We conducted the search in PubMed of the National Library of Medicine using the following keywords: “uterine fibroid”, “leiomyoma”, and “transforming growth factor β”. The aim of this review was to check the available evidence for the influence of the TGF-β family on UF biology. The results of the relevant original studies and reviews published in English up to September 2017 were discussed.

## 2. Discussion

### 2.1. Transforming Growth Factor β—Signaling Pathways, Proliferation and Fibrosis

TGF-β belongs to a much larger family of proteins called the TGF-β superfamily [13,22,26]. This group includes, among others, inhibin, activin, anti-Müllerian hormone, bone morphogenetic proteins (BMPs), and others [13,22,52]. TGF-β controls the proliferation and differentiation of most human cell types and also has proven anti-inflammatory effects. TGF-β is a dimeric polypeptide that is considered to be one of the most important growth factors in the pathogenesis of fibrotic diseases [12,13,22,53].

Various TGF-β isoforms are important in increasing the number of cell divisions in UF tumors and in the overproduction of ECM [12,13,27,53,54]. According to a study by Tang et al., TGF-β induced the proliferation of normal smooth muscle cells [55]. Various researchers found that TGF-β stimulates not only smooth muscle cells, but also causes the proliferation of UF tumors [13,25,56] (Figure 1). Studies on selected cell lines have shown that TGF-β is one of the few cytokines and growth factors that significantly affect the accumulation of ECM in a fibroid tumor [25,29].

TGF-β is secreted as a large latent complex requiring local activation for receptor binding [57]. TGF-β mediates most signals through type I and type II receptors [58]. Type III receptor is a kind of a co-receptor for binding/presenting TGF and a regulator of TGF-β signaling [19]. The active receptor complex phosphorylates Smad proteins, which propagates the signal further [13,14,52,59,60]. The activated complexes will then regulate the transcriptional activity of various genes by many different pathways [19,52,59]. The links in this chain can be as follows: Smad pathway, PI3K/Akt/mTOR, the Ras/Raf/MEK/ERK signaling cascade, and focal adhesion kinase (FAK) [19,52]. The FAK-signaling activated by TGF-β is required for the activation of TGF-β-activated kinase (TAK) [19,61]. The TAK pathway can also be traced to p38 mitogen-activated protein kinases (p38) and nuclear factor κ-light-chain-enhancer of activated B cells NF-κB signaling [62] (Figure 1).

When talking about UF, it is important to mention Wnt signaling, a group of signal transduction pathways that are made of proteins which pass signals into a cell [63]. Wnt signaling pathways are activated by binding a Wnt-protein ligand to a Frizzled family receptor, which passes the biological signal to the specific protein inside the cell. This kind of signaling leads to the regulation of gene transcription [24,63]. The Wnt–β-catenin pathway plays one of the major roles in the functioning of the myometrium and UFs [24]. Certain studies have confirmed that β-catenin regulates and stimulates the renewal of stem cells [64]. Wnt–β-catenin and TGF-β pathways influence the clonal formation of UF tumors and their growth [2,65,66]. Tanwar et al., found that ovarian hormones may accelerate tumorigenesis via the described pathways in mouse models [66]. The Wnt–β-catenin pathway has a major influence on the stimulation of TGF-β3 expression [24,66] and TGF-β3 influences cell proliferation and tumor growth [24] (Figure 2).

According to many researchers, TGF-β3 is considered to be the most important TGF-β3 isoform in UF biology [25,29,56,67]. Its elevated serum levels are one of the risk factors for the occurrence of UF tumors [67]. It has also been observed that TGF-β3 slows ECM degradation [18,29]. Latest studies have indicated that TGF-β3 is one of the very few growth factors that are present in UF tumors in much higher concentrations [26,29]. Despite the proven effects of TGF-β on fibrosis, it remains unclear whether TGF-β3 can trigger this process alone or whether it is only one of the intermediate links. Current laboratory tests have proven that TGF-β3 can directly affect the tissue of the normal myometrium and produce the same processes that occur in UFs [27,29].

The role of TGF-β molecules in the pathophysiology of UFs is also supported by increased expression of latent binding protein-1 and fibrillin-1 in comparison to the healthy myometrium [21,68]. Some data also suggest that TGF-β3 secreted by fibroid cells regulates BMP-2 responsiveness. BMPs play a vital role in bone and cartilage development. These proteins are involved in the TGF-β signaling pathway and in cytokine receptor interaction [69], and are one of the proteins that are responsible for endometrial receptivity, e.g., BMP-2 and BMP-7 [70,71]. TGF-β3 decreases human endometrium receptivity by decreasing the expression of BMP receptors [65]. Sinclair et al., discovered that TGF-β3 may also affect the proliferative effect of prolonged menstruation by acting on BMP-2 [65]. In this study, TGF-β secreted in UF tumors induced BMP-2 resistance in the endometrium by the downregulation of BMP receptor 2, causing defective endometrial decidualization. According to the abovementioned data, this factor was also responsible for the reduced expression of plasminogen activator inhibitor I (PAI-I), thrombomodulin, and antithrombin III, thus contributing to excessive uterine bleeding. Some authors concluded that TGF-β as a single signaling pathway might be responsible for UF-derived infertility and menorrhagia [65].

In a recent publication by Doherty et al., endometrial cells treated with TGF-β3 were shown to have altered BMP-2 responsiveness [70]. This resistance is a major point in impaired decidualization and subsequent infertility [70]. As far as TGF-β is concerned, the main point of interest for the researchers is excessive fibrosis, but this molecule is also responsible for tumor angiogenesis and its pathological influence, including endothelial cell proliferation, migration, and expression of adhesion molecules [44,72].

All of the abovementioned data support the hypothesis that the TGF-β signaling pathway, which contains numerous ligands, receptors, and molecules, might be a potential target of dysregulation and be involved in UF formation, growth, or symptom occurrence. These pathways might also be the key point of future clinical investigations [65].

### 2.2. Extracellular Matrix

The main mechanisms of UF growth include increased ECM production, cell migration to a specific location, stimulation of growth factor expression, and the subsequent deposition of an increased number of abnormal ECM [13,17]. According to latest research, steroid hormones are responsible for the expression of growth factor and cytokine genes in UF molecular pathways, resulting in an increased number of cell divisions and ECM production, which in turn leads to further tumor growth [17,24,34].

One of the most important features of UFs is the abundance of fibrotic connective tissue and ECM components, with excessive production of type I collagen, fibronectin, and glycosaminoglycans. These molecules also have incorrect spatial architecture [17,34,73]. In UFs, fibronectin expression is particularly enhanced, which is bound to the pathways that depend on TGF-β [27]. According to Norian et al., disturbed production of disorganized ECM occurs largely due to activation of the pathway dependent on transforming growth factors and overproduction of glycosaminoglycan-rich versican variants [34].

MMPs are calcium-dependent endopeptidases which contain zinc cations in their structure. They essentially serve to degrade and rebuild the ECM structure [74]. Studies have shown that MMPs and their mRNAs are increasingly expressed in UFs [75,76]. During laboratory tests, TGF-β has been shown to reduce the concentration of the corresponding MMPs. TGF-β also increases the concentration of their inhibitors, which slows down the conversion of the entire ECM of the UF, and results in excessive ECM accumulation [27,77,78]. Slowed down by high concentrations of TGF-β, MMPs cannot degrade a sufficient amount of ECM to maintain balance in the tissue [74,78]. The TGF-β3 isoform has a particular role in this process, which has been confirmed by numerous studies [14,29]. Halder et al., found that vitamin D inhibits the expression and activities of selected MMPs in UF cells by influencing TGF-β3 [27].

Out of all three isoforms, the TGF-β3 isoform has the greatest role in the ECM overproduction by stimulating the expression of type I collagen, fibronectin, laminin, and proteoglycans [17,34,35,54]. UF cells in laboratory studies demonstrated an increase in TGF-β3 mRNA expression as compared to healthy smooth muscle [29,34], which in turn induces increased ECM secretion in the uterine myometrium [77] (Figure 2).

### 2.3. Regulation by Steroids

The development of UFs is multifactorial in nature. According to various studies, UF growth depends mostly on steroid hormones [24,36,37]. The effects of estrogen and progesterone on these tumors are numerous [24]. Most authors confirm that long-term stimulation of the myometrial cells by estrogen and progesterone leads to the formation and growth of UFs [1,24] (Figure 3). Based on the available data, it can be expected that progesterone, instead of estrogen, plays the key role in the process of UF formation [14,24]. The luteal phase is the time when higher levels of progesterone receptors are found in the tissues. In the case of UFs, this is associated with the inhibition of apoptosis and the acceleration of growth [24,39]. An interesting observation related to TGF-β and UFs is that the highest concentrations of TGF-β mRNA are observed in the secretory phase of the menstrual cycle [56]. It confirms that progesterone is primarily bound to this factor and affects the expression of selected genes [56]. Thus, it can be concluded that the effect of progesterone on UF growth is determined by the overexpression as well as increased concentrations of various growth factor genes (including TGF-β) [24,36]. Subsequent studies have confirmed elevated growth factor levels both, peripherally as well as in the tumors themselves [13,17,24,79]. The positive effect of progesterone on UF growth is confirmed by the use of its antagonists in the treatment of UFs [46].

While taking into consideration the evidence for the effect of progesterone on UF, it is advisable to bear in mind the role of estrogens, which, despite their smaller role, are in fact preparing the tumor to be stimulated by progesterone by upregulating progesterone receptors [14,24]. An overexpression of the estrogen receptor in UFs is observed, as compared to normal smooth muscle tissue of the uterus. UF cells exhibit excessive sensitivity to estrogens as compared to normal muscle [36,39,80,81]. The estrogen receptor α signaling pathway has an influence on the TGF-β signaling pathway under the effect of estrogen and other similar molecules [82]. Studies in UF cell colonies showed that the proliferative potential can be acquired by tumor tissue through estrogen stimulation. Under the influence of estrogens, UFs remained more active in the subdivisions, and their apoptosis slowed down. Despite these data, the reduced apoptotic potential, along with increased proliferative potential, is associated more with the progesterone component than estrogen [83,84] (Figure 3).

### 2.4. Genetics

According to Makinen et al., specific mutations were detected within the gene encoding the MED12 located on the X chromosome in the examined UFs [43]. The mediator complex is a 26-sub transcript regulator that is essential for proper transcription. The mediator complex is highly conserved in eukaryote organisms [24,85]. All of the *MED12* gene mutations are located within exon 2 and are probably responsible for the mechanism of tumorigenesis [43]. Further studies have shown that mutations within exon 2 may occur even in 85% of UF-positive patients, depending on the population [86,87,88]. Mutations in *MED12* are also present in other mesenchymal tumors of the uterus or in other tissues [89]. MED12 is linked to β-catenin and regulates Wnt signaling [24,90]. A study confirmed that Wnt expression is elevated in UFs in the case of mutations within the *MED12* gene [91]. A recent study by Al-Hendy et al., suggests that the silencing of the *MED12* gene reduces the proliferation of UF tumor cells by the Wnt-β-catenin signaling pathway [92].

The reasons for our interest in that topic are numerous. MED12 deficiency activates the TGF-β pathway, utilizing two types of signaling: Smad and mitogen-activated protein kinase (MAPK) related [24,93]. Smads are intracellular proteins which transduce extracellular signals from TGF-β ligands to the nucleus [20,94]. MAPK is a type of protein kinase that is involved in directing cellular responses to different stimuli. MAPK regulates cell functions, including proliferation, gene expression, differentiation, and apoptosis [95]. The TGF-β activation induced by this path results in further signaling and has the effect of renewing stem cells, cell growth and division, and fibrosis [24].

### 2.5. TGF-β and Implications for Therapy

The description of the above relationships confirms the assumption that pathways are dependent on estrogen and progesterone, and thus TGF-β has a tremendous effect on the way stem cells are divided and affects their conversion into clonal cells, which create UFs [24,14].

According to Tal et al., the growth of UF tumors is dependent on steroids partly due to their induction of local angiogenic factors for the provision of new vessels [44]. Shen et al., who investigated how uterine artery embolization influences UF tumor blood supply, observed that tumor diameter was significantly lower than before treatment, and that the TGF-β level was significantly decreased [96].

GnRHa (e.g., leuprolide) has been observed to effectively reduce both, UF growth and the accompanying symptoms that are TGF-β dependent [93]. In vitro studies involving the administration of GnRHa to cell cultures have confirmed inhibition of the synthesis of UF DNA under the influence of these drugs [97]. There are other sources that confirm that GnRHa are effective in reducing the expression of the TGF-β family proteins and their receptors by causing a menopause-like condition [13,98,99]. The available studies demonstrated that GnRHa treatment results in decreased expression of many cytokines, including the TGF-β family, as well as reduced tumor volume [44,97] (Figure 3).

Similarly, AIs reduce the amount of active hormones that affect UFs. AIs are a class of drugs that present the antiestrogenic effect. The most well-known AIs include anastrozole, letrozole, and fadrozole. They are mainly used in the treatment of gynecological cancers [100]. Their efficacy in UF treatments has also been confirmed [84] (Figure 3).

The abovementioned therapies are currently not commonly used because of ulipristal acetate (UPA), a type of SPRM that has become the primary drug in the treatment of UFs in selected indications [46,101]. The positive influence of progesterone on UF growth is implied by the efficacy of its antagonists in pharmacological therapy. The advantages of UPA include its large information base, good safety profile, and good tolerance [46,102]. Numerous clinical studies have confirmed its effectiveness [46,102,103,104,105], and ongoing studies will define new treatment regimens. UPA affects the progesterone receptor, which may affect the reduction of TGF-β production (as described above), followed by inhibition of fibrosis and fibroid growth, and is a likely pathway for its action (Figure 3). Other laboratories (including our own) are currently conducting research to determine the effect of UPA on TGF-β levels in serum and in UF tissue, but more data are required.

### 2.6. Future Ideas

Cell studies have shown that vitamin D reduces the expression of steroid receptors in UF cells in laboratory conditions, which may have important clinical implications and be one of the determinants of the pathogenesis and pathological growth of UFs [106]. Multiple studies have shown that vitamin D induces apoptosis of UF cells, lowers the TGF-β pro-fibroid effect, and modulates the expression of MMPs and TIMPs [27,49,77]. TGF-β3 was inhibited by increased levels of vitamin D [35]. Animal studies have shown that the administration of therapeutic doses of vitamin D3 significantly reduces UF size [107].

As far as UF treatment is concerned, vitamin D remains the best-studied alternative substance that affects UFs through pathways dependent on TGF-β. However, it would be prudent to remember about paricalcitol, which is an analog of vitamin D. It has been proven that paricalcitol effectively reduces the proliferation of human leiomyoma cell cultures, reduces fibroid tumor volumes, and induces apoptosis in UFs [108]. This agent has great potential as an effective drug in this disease, but after several randomized controlled trials.

SB525334, a potent and selective inhibitor of TGF-β receptor I (ALK5), is yet another interesting substance that has been very poorly examined so far [109]. This substance has not been widely tested in the treatment of UFs. According to the study by Laping et al., treatment with this agent decreases the incidence, number, and size of UF tumors in a mutant rodent model [110]. More data on how SB525334 affects other diseases, such as kidney or lung fibrosis, are necessary [109]. However, due to previously mentioned examples regarding the role of TGF-β in UF biology, SB525334 and its derivatives might herald a new high-quality treatment in UF therapy [111].

There are studies on the use of nonsteroidal anti-inflammatory drugs, such as celecoxib, a cyclooxygenase 2 inhibitor, as a cytokine-reducing agent that is necessary for UF growth [112]. In a study by Park et al., celecoxib decreased the gene expression of several cytokines, including TGF-β [112]. This study suggests that celecoxib could inhibit the growth of UFs by blocking the inflammatory pathway, but further research is needed to confirm the effects of celecoxib on UF growth.

There is also some data about the use of tranilast in UF therapy. Tranilast inhibits the rate of cell growth, TGF-β-derived collagen biosynthesis, growth factor expression, and fibroblast transformation [12,113]. Tranilast has a direct effect on UF cells through the altered expression of miR-29c and genes functionally involved in cell cycle progression and tissue fibrosis and might have a therapeutic potential as an inhibitory agent for UF growth and clinical symptom regression [114].

All of these new agents, although still under evaluation and are not available as registered treatments, use pathways that are related to the TGF-β family to treat UFs. They provide unique benefits for potential effectiveness alone or when used as co-drugs with drugs like SPRMs or AIs. We hope that in the future we will be able to provide multivalent therapies that will be extremely effective, safe, and individually tailored to patient characteristics.

## 3. Conclusions

Growth factors are one of the key players in the development and proliferation of UFs. The TGF-β family is one of the most important regulators of the fibrosis processes. A large number of studies have confirmed that it has a significant effect on the development and growth of UFs. Abnormal concentrations or overexpression of TGF-β mediators may also be responsible for some of the clinical symptoms that are associated with TGF-β, especially in cases of the TGF-β3 isoform. TGF-β and its dependent processes in UFs are mostly regulated through steroid hormones, which are reflected in the available therapies. Currently, UPA has become the gold standard in UF therapy, but there are also nonsteroidal substances that affect the pathways dependent on TGF-β, which may, after extensive research, also become useful tools in the treatment of UF tumors.

## Abbreviations

AI	Aromatase inhibtor
Akt	Protein kinase B
ALK	TGFβ type I receptor kinase
BMP	Bone morphogenic protein
ECM	Extracellular matrix
ER	Estrogen receptor
ERK	Extracellular signal-regulated kinases
FAK	Focal adhesion kinase
GnRH	Gonadotropin releasing hormone
GnRHa	Gonadotropin releasing hormone agonist
JNK	c-Jun N-terminal kinase
MAPK	Mitogen-activated protein kinase
MED12	Mediator complex subunit 12
MEK	MAPK/ERK kinase
MKK	Mitogen-activated protein kinase kinase
MMP	Matrix metalloproteinase
mRNA	messenger RNA
mTOR	mechanistic target of rapamycin
NF-κB	nuclear factor kappa-light-chain-enhancer of activated B cells
p38	p38 mitogen-activated protein kinases
PI3K	Phosphoinositide 3-kinase
PR	Progesterone receptor
Raf	Raf protein
Ras	Ras protein
Smad	Smad protein
SPRM	Selective progesterone receptor modulator
TAK	Transforming growth factor β-activated kinase
TGF-β	Transforming growth factor β
TGF-βR	Transforming growth factor β receptor
TIMP	Tissue inhibitor of matrix metalloproteinase
UF	Uterine fibroid
